# UTILIZING ADVANCED DRIVER ASSISTANCE SYSTEMS IN LATER LIFE: INSIGHTS FROM A LONGITUDINAL STUDY ON AGING DRIVERS

**DOI:** 10.1093/geroni/igad104.3690

**Published:** 2023-12-21

**Authors:** Renee St Louis, David Eby, Lidia Kostyniuk, Lisa Molnar, Jennifer Zakrajsek, Nicole Zanier, Linda Nyquist, Raymond Yung

**Affiliations:** University of Michigan Transportation Research Institute, Ann Arbor, Michigan, United States; University of Michigan, Ann Arbor, Michigan, United States; University of Michigan Transportation Research Institute, Ann Arbor, Michigan, United States; University of Michigan, Ann Arbor, Michigan, United States; University of Michigan, Ann Arbor, Michigan, United States; University of Michigan Transportation Research Institute, Ann Arbor, Michigan, United States; University of Michigan Geriatrics, Ann Arbor, Michigan, United States; University of Michigan Geriatrics, Ann Arbor, Michigan, United States

## Abstract

Level 1 and 2 Advanced Driver Assistance System (ADAS) technologies (e.g., adaptive cruise control, blind spot warning, lane keep assist) are predicted to be available in approximately three-fourths of all vehicles worldwide by 2025. To realize the potential of these systems in extending safe mobility for aging adults, it is critical that the driver understands the functionality of the technology and uses the system appropriately. The current study examined demographic and vehicle technology questionnaire data from the Longitudinal Research on Aging Drivers (LongROAD) cohort study which concluded in December 2022. A total of 1,417 participants changed their vehicle throughout the study. There were statistically significant increases in the prevalence of all 15 ADAS technologies examined. Despite increases in prevalence, frequency of using the technologies remained unchanged across 5 years. Frequency of use also varied by functionality of the technology whereby participants reported higher frequency of using technologies that provide alerts, such as blind spot warning, than technologies that take action to assist drivers with vehicle operations, such as adaptive cruise control. Results showed differences in prevalence and use of technologies by income and education, suggesting disparities in access to vehicles with technologies that could help to create a safer driving experience. In consideration of the rapid proliferation of ADAS into the vehicle fleet, increased research into how older drivers learn about and use ADAS technologies will assist in efforts to develop tailored and accessible programs for training older adults to properly utilize ADAS available in their own vehicles.

